# Transfer of Human Microbiome to Drosophila Gut Model

**DOI:** 10.3390/microorganisms10030553

**Published:** 2022-03-03

**Authors:** Dongxu Ji, Hao Sun, Weichao Yang, Mingfu Gao, Hui Xu

**Affiliations:** 1 Key Laboratory of Pollution Ecology and Environmental Engineering, Institute of Applied Ecology, Chinese Academy of Sciences, 72 Wenhua Road, Shenyang 110016, China; jidongxu18@mails.ucas.ac.cn (D.J.); yangweichao@iae.ac.cn (W.Y.); mingfujun@163.com (M.G.); 2 College of Resources and Environment, University of Chinese Academy of Sciences, Beijing 100049, China

**Keywords:** gut microbiota, fecal microbiota exposure, *Drosophila melanogaster*, antibiotic

## Abstract

Laboratory animals with human microbiome have increasingly been used to study the role of bacteria and host interaction. *Drosophila melanogaster*, as a model of microbiota-host interaction with high reproductive efficiency and high availability, has always been lacking studies of interaction with human gut microbiome. In this study, we attempted to use antibiotic therapy and human fecal exposure strategy to transfer the human microbiome to the drosophila. The method includes depleting the original intestinal bacteria using a broad-spectrum antibiotic and then introducing human microorganisms by a diet supplemented with donor’s fecal samples. The sequencing results showed that 80–87.5% of the OTUs (Operational Taxonomic Units) from donor feces were adopted by the recipient drosophila following 30 days of observation. In comparison to females, the male recipient drosophila inherited more microbiota from the donor feces and had significantly increased lifespan as well as improved vertical climbing ability. Furthermore, distinctly differential expression patterns for age and insulin-like signaling-related genes were obtained for the male vs. female recipients. Only the male drosophila offspring acquired the characteristics of the donor fecal microbiota.

## 1. Introduction

The intestinal bacteria comprise the microbial community that resides in the human intestine. The number of bacteria in the human intestine is approximately 100 trillion, with the corresponding total number of genes approximately 150 times the number of human genes. Therefore, intestinal bacteria are also known as the “second genome” of the human body [1]. It has been confirmed that intestinal bacteria play a key role in most metabolic processes and are associated with many diseases [2,3,4].

Experimental animals are the main tools for intestinal microbiota studies and they are used to investigate aspects of pathology, pharmacology, metabolism, and pharmacokinetic kinetics. Currently used model organisms include nematodes, drosophila, zebrafish, and mice, but the intestinal bacterial communities of these experimental animals are still very different from that found in humans, which limits advanced research and development in this area based on the use of these models. The emergence of humanized mice has made the study of disease phenotypes and intestinal humanization possible [5,6,7]. Traditionally, the establishment of the model has involved inoculating germ-free mice with bacteria from human donors to provide a feasible system for use in a study that is not suitable for human subjects. However, the most common and/or most important systems used in research include many proximal intersections and genetic murine models, and sterile mice are not easy to obtain [8]. In addition, the development of humanized murine models is time-consuming and expensive. Therefore, there is an urgent need for a new experimental model in which to examine human intestinal microbiota.

*Drosophila*, as a model organism, has many advantages. First, there is a high degree of homology between the sequences of the drosophila and human genomes, which further confirms the potential of drosophila as a model in which to study human diseases [9,10]. Functional drosophila homologs can be found in the case of approximately 75% of the genes associated with human diseases [11]. Second, drosophila has a relatively short life cycle and fast reproductive rate. Normally, fertile drosophila mated at around 25 °C can produce hundreds of genetically identical offspring in approximately 10 days, which is significantly faster than the rodent models typically used. In recent years, drosophila have also been used to study the relationship between intestinal bacteria and diseases [12,13,14,15]. The digestive tract of the drosophila, in comparison to that of humans, is relatively conservative and can be divided into foregut, midgut, and hindgut segments. Moreover, the mechanisms that limit the contact between the intestinal microbiota and the intestinal epithelial cells in mammals are also conserved in drosophila, including the acidic regions (e.g., mammalian stomach and copper cells), the mucus secretion that forms a protective mucous layer, and the secretion of antimicrobial peptides [16]. However, compared to humans and rodents, the drosophila gut microbial structure is much simpler with only 1–30 species and the dominant bacteria are also very different in terms of taxonomy, including mainly *Lactobacillus* and *Acetobacter* [12,17,18].

To the best of our knowledge, the humanization of drosophila gut bacteria has not been investigated to date. To successfully construct a drosophila intestinal model with human microbiome for high-throughput in vivo studies of the human intestinal microbiome, the aforementioned shortcomings would need to be addressed. The transfer of human microbiota to drosophila may be possible based on the following: (1). the interaction between the host drosophila and their intestinal bacteria is similar to that found in humans, wherein the beneficial bacteria have a mutually beneficial relationship with their host [19]; (2). microorganisms obtained from the environment shortly after drosophila are born can survive in the intestinal tract and exhibit stable existence during their life cycle [16]. We speculated that this feature combined with fecal microbiota exposure could make it possible to transfer human microbiota to drosophila; and (3). drosophila consume manure as a food source, which stabilizes the structure of the intestinal bacteria between the drosophila parents and their offspring.

In this study, we attempted to develop a convenient and anthropomorphic drosophila model of human intestinal bacteria that can be used for scientific research. To establish such a model, we first transfer human microbiota to drosophila by using antibiotic therapy and human fecal microbiota exposure, and we then investigated the stability of the intestinal bacteria and its heritability of passage through the analysis of 16S rDNA sequencing results.

## 2. Materials and Methods

### 2.1. Collection and Processing of Fecal Samples

**Figure 1:**
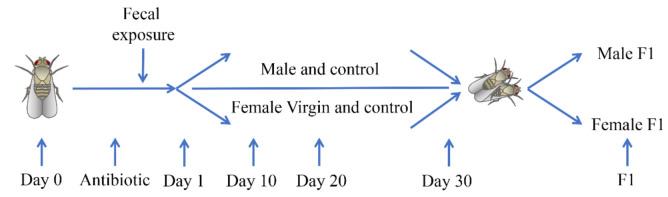
Diagram showing details of the test process and the date of each sampling. We first selected female virgin flies and male flies within 8 h (recorded as Day 0), treated them with antibiotics for 5 days (chlortetracycline concentration: 50 μg/mL), and then exposed them to fecal microbiota (recorded as Day 1). F1 is the offspring of fruit flies produced by mating female virgin flies and male fruit flies after 30 days of fecal microbiota exposure, and F1 flies were cultured on a standard medium.

1. Preliminary work: firstly, a suitable donor was screened according to the Brandt induction method and the donor questionnaire was filled out by the parents of the donor (as the donor was a two-year-old boy) [20]. Subsequently, the donor underwent comprehensive blood and fecal screening to detect potential transmissible pathogens. The donor’s parents were instructed not to administer any medications for at least one week before and during the fecal samples collection period as donors should not be treated by antibiotics for eight weeks prior to feces collection.
2. Sample collection: we instructed the donor’s parents to collect the fecal samples in the collection cap according to the standard operating procedure for feces collection and then label the cap with the time and donor information after sealing. Urine, paper, or other waste were kept away from the sample during this process.
3. Sample transportation and preservation: the fecal samples were maintained at 2–8 °C during transportation and then stored at −20 °C in the laboratory.
4. Sample processing: weigh 50 g of uncontaminated fecal samples, add 300 mL of sterile water to it, stir well, filter with 4 × 4 sterile gauze, and then collect the filtrate to add to the drosophila culture medium. Details of the test process and the date of each sampling please see Figure 1.

### 2.2. Drosophila Husbandry

We procured *Drosophila melanogaster* from Bloomington Drosophila Stock Center (wild type #9516, Indiana University, Bloomington, IN, USA) for experiments. The flies were raised on a standard diet, which contained 1 g sodium benzoate, 9.18 g soybean meal, 0.25 g nipagin, 40 g sucrose, 25 g yeast, 66.825 g cornmeal, 42.4 g maltose, 6 g agar, and 6.875 mL propionic acid per liter.

Culture medium (containing antibiotic): a filtered solution of chlortetracycline (CT) (Macklin, Shanghai, China, C822258) at a dilution rate of 1/20 (final concentration: 50 μg/mL) was added to drosophila standard culture medium and thoroughly mixed before the culture medium solidified.

Fecal culture medium: a 100 μL aliquot of fecal suspension filtrate was added into 5 mL drosophila standard culture medium.

We first selected female virgin flies and male flies within 8 h (recorded as Day 0), treated them with antibiotics for 5 days, and then exposed them to fecal microbiota (recorded as Day 1). All flies were maintained under a constant temperature (25 °C) and humidity (65%) with a 12 h light-dark cycle.

### 2.3. Lifespan Assay

Under mild anesthesia using an appropriate concentration of carbon dioxide, the drosophila that were incubated within 8 h were divided into females and males [21]. All of the female drosophila were virgin flies. Ten vials, each containing 20 drosophila, were used for survival analysis. The survival curve was determined by counting the dead drosophila every 2–3 days, and the medium was renewed every 3–4 days. In each case, 8 vials containing 20 drosophila were counted.

### 2.4. Body Weight

On days 10, 20, and 30, the body weights of the examined drosophila were recorded. The drosophila were anesthetized with an appropriate concentration of carbon dioxide. Afterward, their body weights were measured using an ultra-sensitive scale (Sartorius, Beijing, China).

### 2.5. Locomotor Activity

To assess the activity of drosophila, the vertical climbing ability of drosophila was measured. Twenty drosophila were placed on the bottom of a clean 4-inch glass bottle and another identical bottle was placed on top so that their respective openings formed a weak seal. After 30 s, the two vials were separated [22,23,24]. The drosophila climbing indices were expressed as the percentage of the number of drosophila that could climb to the upper bottle to the total number of tests. On day 30 drosophila were selected and performed three climbing tests in each bottle. Wilcoxon rank-sum and ANOVA tests were used for statistical analysis.

### 2.6. 16S rDNA Amplicon Sequencing and Analysis

16S rDNA amplicon sequencing was performed to explore the position and shifts of the gut microbiota in the tested drosophila. Firstly, drosophila were rinsed in 50% bleach (sodium hypochlorite), 70% ethanol, and sterile PBS prior to dissection. Each fly was dissected in sterile PBS, and crops, Malpighian tubules, and trachea were removed. The dissected midgut was collected and homogenized, and DNeasy Blood and Tissue Kits (Qiagen, 69504, Hilden, Germany) were used to extract genomic DNA. Hypervariable regions V3–V4 of prokaryotic 16S rDNA were amplified by using primers (“3′-CCTACGGRRBGCASCAGKVRVGAAT-5′” and “5′-GGACTACNVGGGTWTCTAATCC-3′”). During this step, indexed adapters were also added to the ends of the amplicons to generate indexed libraries. The concentration of DNA in the libraries was quantified using a Qubit 3.0 fluorometer, adjusted to 10 nM, and the DNA libraries were then loaded on an Illumina MiSeq instrument for multiplex analysis (Illumina, San Diego, CA, USA).

In double-ended sequencing of positive and negative reads, the first of the two pairs are joined together to filter according to the results contained in the N terminal sequence, whereby sequences of lengths longer than 200 bp are retained.

The chimeric sequences that remained after quality filtering and purification were clustered using VSEARCH (1.9.6) where the sequence similarity was set to 97%, according to the database Silva 132 [25]. Subsequently, the Ribosomal Database Program (RDP) was used to classify the OTUs (Operational Taxonomic Units).

Downstream analysis was performed with phyloseq (V 3.14) and microeco (v0.6.0, https://github.com/ChiLiubio/microeco, accessed on 16 November 2021) packages in R (V 4.12), which included α diversity indices to calculate dimensionality reduction and ranking analysis. Nonmetric multidimensional scaling (NMDS) was performed according to the bacterial Bray–Curtis distance matrix, which displayed the β diversity. We also calculated the abundance-based Bray–Curtis dissimilarity as the metrics of β-diversity to quantify community compositional difference between replicate plots of the same treatment. Linear discriminant analysis (LDA) and linear effect-size (LEfSe) analyses were conducted using the online Galaxy website. The RAW reads obtained from this study were deposited into the NCBI SRA database under the accession number PRJNA787037.

### 2.7. Quantitative Real-Time PCR

Total RNA was extracted from 15–20 drosophila samples on days 10, 20, and 30. The FastKing First-Strand Synthesis system (Tiangen, KR116-02, Beijing, China) was used to reverse-transcribe 1 µg of total RNA to cDNA according to the manufacturer’s instructions. QRT-PCR (Quantitative Real-Time Polymerase Chain Reaction) was performed with QuantiNova SYBR Green I PCR Master Mix (Qiagen, 208052, Hilden, Germany). The primer sequences and programs are included in Supplementary Tables S1 and S2. Three technical replicates of three biological replicates were used in the analysis of each sample, and the final mRNA expression dynamics relative to the controls were normalized to the housekeeping mRNA that encodes rp49 [19,26].

### 2.8. Statistics

The drosophila survival curves and Mantel–Cox tests were analyzed using GraphPad Prism 8 software (GraphPad Software, La Jolla, CA, USA). For multiple comparisons, one-way analysis of variance with Tukey’s test was used; asterisks indicate statistical significance, where * *p* < 0.05 and ** *p* < 0.01.

## 3. Results

### 3.1. Dynamics of α-Diversity and Bacterial Composition of Gut Bacteria

**Figure 2:**
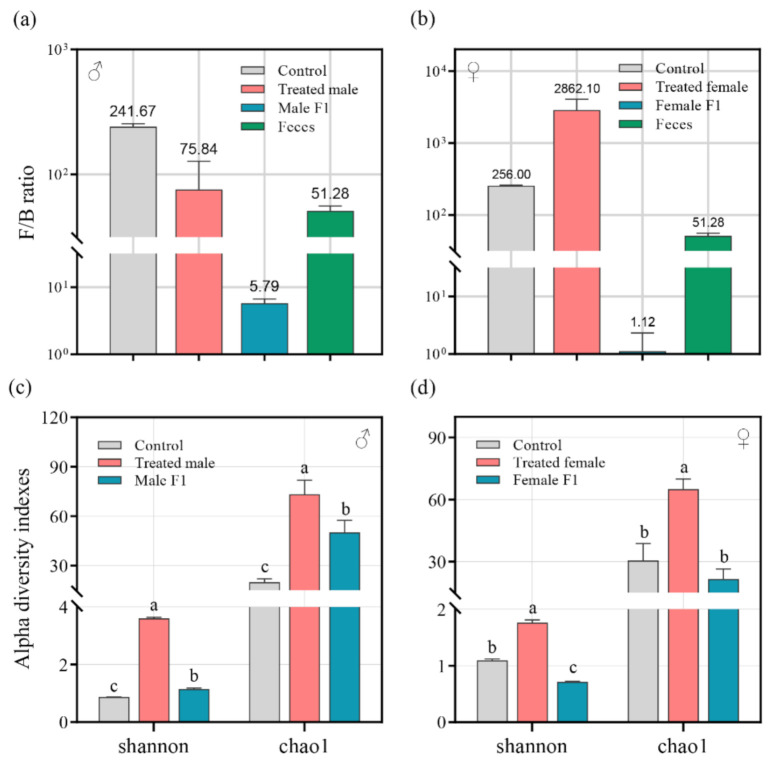
(**a**,**b**) The Firmicutes/Bacteroidetes ratio (F/B ratio) in male (**a**) and female (**b**) drosophila under fecal microbiota exposure; (**c**,**d**) Shannon and Chao1 indices indicated gut bacterial diversity in male (**c**) and female (**d**) drosophila. Samples were taken from drosophila cultivated for 30 days.

After the antibiotic and fecal treatment, the dynamics of the Firmicutes/Bacteroidetes ratio (F/B ratio) showed significant differences between sexes. As compared to that of CK (Control Check) (average 241.67), the F/B ratio in the treated male group (average 75.84) and the male F1 group (average 5.79) were both dramatically reduced (*p* < 0.01) (Figure 2a). The F/B ratio in the female F1 group was also significantly reduced (average 1.12 in female F1 vs. 256 in CK, *p* < 0.01) (Figure 2b); however, the F/B ratio of the treated female group increased (average 2862.1 in female F1 vs. 256 in CK, *p* < 0.01).

**Figure 3:**
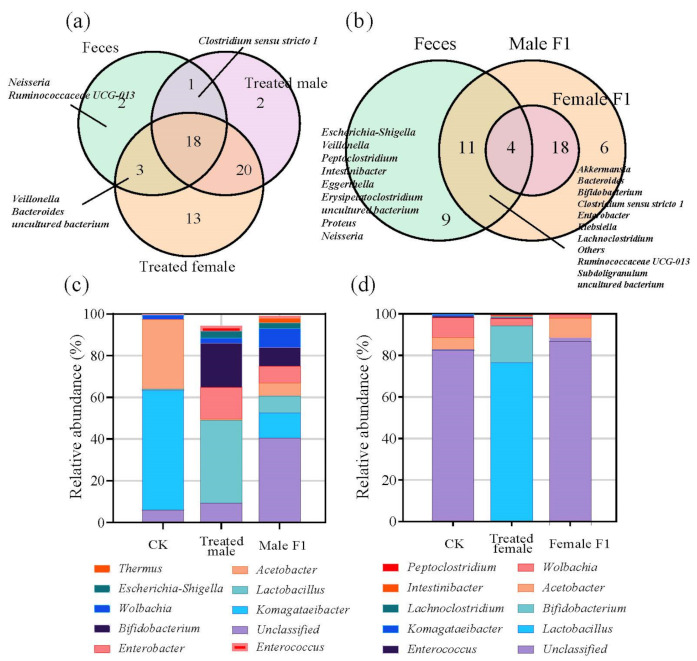
(**a**,**b**) Venn diagram of genus-level species abundances of the treatment group (**a**) and the F1 group (**b**); (**c**,**d**) top 20 species in male (**c**) and female (**d**) drosophila in relative abundance at genus level. Samples were taken from drosophila cultivated for 30 days.

Totally, the sequencing analysis showed that 80–87.5% of OTUs that had belonged to the donor feces were adopted by the recipient drosophila during the 30 days of observation (Figure S1). It is also showed that the treated drosophila adopted 79.17–87.5% of represented taxa at the genus level from the microorganisms of the donor feces on day 30 of observation (Figure 3a); among these, male treated samples adopted 79.17% fecal genera (19 of 24 fecal genera), while the female treated samples adopted 87.5% fecal genera (21 of 24 fecal). Inheritance rates decreased in progeny flies that were not exposed to feces, the male F1 group adopted 62.5% (9 of 24 fecal genera), while the female F1 group adopted 16.67% genera (4 of 24 fecal genera) (Figure 3b). Uninherited fecal bacterial genera in each sample were listed in Table S3. The exposure to fecal microbiota resulted in a large enrichment of bifidobacteria and lactobacilli in the treated male group (i.e., bifidobacteria 21.11% and lactobacilli 39.50%), as well as the treated female group (i.e., bifidobacteria 17.56% and lactobacilli 76.20%) (Figure 3c,d). In addition, the exposure to fecal microbiota enabled the male F1 group to enrich the taxa including *Akkermansia*, *Bacteroides, Clostridium sensu stricto* 1, *Enterobacter*, *Klebsiella*, and *Ruminococcaceae* UCG-013, which do not exist in wild-type drosophila (Table S4).

The Chao1 and Shannon indices were used to analyze the α diversity of the samples as well as the impact of exposure of the drosophila to fecal microbiota. The results show that, compared to the control group, the richness and the uniformity of the species in the treated male group (average Chao1 of 73.4 vs. 20 (CK) and average Shannon of 3.61 vs. 0.87 (CK)), the male F1 group (average Chao1: 50.17, Shannon: 1.14), and the treated female group (average Chao1 of 65.02 vs. 30.5 (CK) and average Shannon of 1.76 vs. 1.09 (CK)) had an identifiable improvement (Chao1 index: *p* < 0.01; Shannon index: *p* < 0.01) (Figure 2c,d), but a sharp reduction in the Shannon index occurred in the female F1 group (average Shannon: 0.72 vs. CK with average Shannon: 1.09, *p* < 0.01) (Figure 2d).

### 3.2. Analysis of Gut Bacterial Similarity

**Figure 4:**
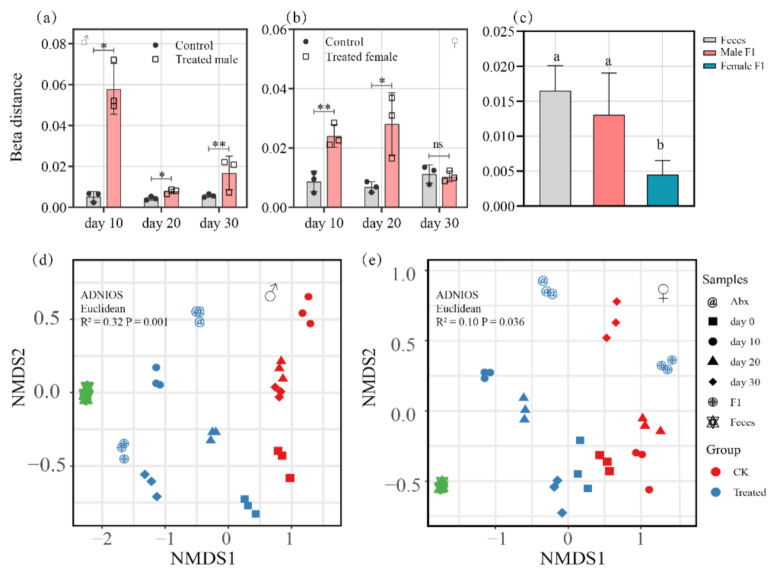
(**a**,**b**) Beta distance of bacterial community of the treated male (**a**) and female (**b**) drosophila; (**c**) beta distance of bacterial community of the feces and drosophila F1 (samples were taken from drosophila cultivated for 30 days), * *p* ≤ 0.05; ** *p* < 0.01; (**d**,**e**) nonmetric multidimensional scaling (NMDS) analysis based on weighted UniFrac metrics shows shifts in the bacterial communities driven by the fecal microbiota in male (**d**) and female (**e**) drosophila.

As compared with the control group, the beta distance of the treated male group showed notable differences on day 10, 20, and 30 of sampling (10d, *p* < 0.01; 20d, *p* < 0.05; 30d *p* < 0.01) (Figure 4a). However, the treated female group had only a subtle distinction on the 30th sampling day (*p* = 0.70) (Figure 4b). This indicates that fecal exposure drove a sex-biased change in the gut microbe structures in drosophila.

The results also show that there were no dramatic differences in beta distance between the bacterial communities of the male F1 group and the feces (*p* = 0.44) (Figure 4c), which indicated that the bacterial community of the male F1 group is similar to that of the fecal samples. However, the bacterial communities of the female F1 group and the feces were found to be significantly different based on beta distance (*p* < 0.01) (Figure 4c).

Through NMDS analysis, we found that, as a result of the fecal microbiota, the bacterial community of the treated male group and the control group could be divided into two different groups on Axis.1 (*p* = 0.001) (Figure 4d). Meanwhile, there was a remarkable difference between the bacterial community of the treated female group vs. the control group (*p* = 0.036) (Figure 4e). The results indicate that there was a significant change in the intestinal bacteria of the male and female drosophila of the treatment group, but that fecal exposure had a more pronounced impact on the intestinal bacteria of the male than of the female drosophila, and the influence was even extended to the offspring of the male group.

### 3.3. Effects of Fecal Microbiota Exposure on Species with Significant Differences in Abundance and Community Structure

**Figure 5:**
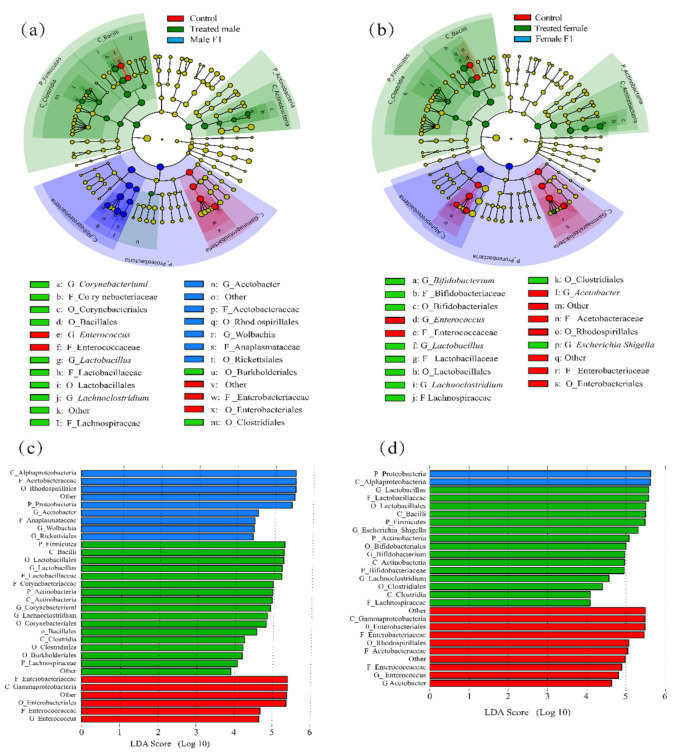
(**a**,**b**) An evolutionary clade of the male (**a**) and female (**b**) drosophila under fecal microbiota exposure; (**c**,**d**) LDA scores for microbes differentially abundant between control, treated, and F1 samples of male (**c**) and female (**d**) drosophila. (Treated samples were taken from drosophila cultivated for 30 days).

First, by using LEfSe analysis, we found that, as a result of the exposure to the fecal microbiota, distinct evolutionary clusters formed in the treatment group (Figure 5a,b); LDA was used to determine the OTU based on the threshold values and to show the changes in species with differential values. *Lactobacillus* was the dominating bacterial species in donor feces. Our result shows *Lactobacillus* played a vital role in both the treated male and treated female groups. Firmicutes and Actinobacteria become the differential bacterial species in both the treated male and treated female groups at phylum level, which indicates that the treated flies obtained the fecal donor’s microbiota.

Alphaproteobacteria (at class level) were the most differential species in the female F1, while Acetobacteraceae (at class level) species accounted for a large proportion in the male F1 group. These two species are included in the significantly differential species in the control group at the phylum level (Proteobacteria) (Figure 5a,b).

At genus level, the differential abundance of taxa in the male control group was *Enterobacteriales*; and in the treated male group included *Lactobacillales* and *Lachnoclostridium*; and in the male F1 group were *Acetobacteraceae*, and *Wolbachia*, (Figure 5c). In the treated female group, most differential bacterial species includes *Bifidobacterium, Lactobacillales*, *Lachnoclostridium*, and *Escherichia shigella*. While those in the female F1 were *Enterobacteriales and Acetobacter* (Figure 5d). It was worth noticing that *Escherichia shigella,* which enriched in the treated female group, was an obligate agent of humans. By day 30 the relative abundance of *Escherichia shigella* in the treated female group was averagely 3.47% (0.41% in feces sample). It is indicated that *Escherichia shigella* was stably colonized and proliferated in female drosophila.

### 3.4. Effects of Fecal Microbiota Exposure on Drosophila Lifespan and Body Weight

**Figure 6:**
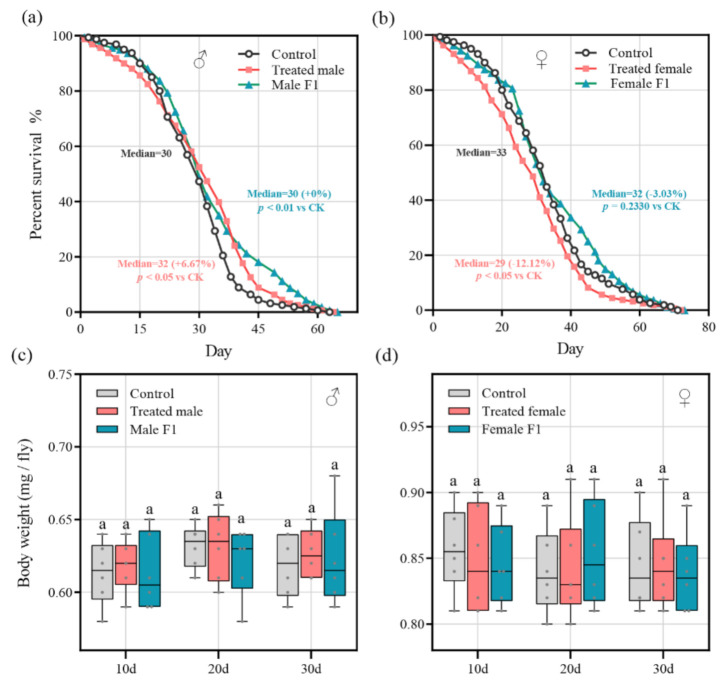
(**a**,**b**) Lifespan curves show how the treatment impacted the lifespan of the male (**a**) and female (**b**) drosophila; (**c**,**d**) the box plot shows the bodyweight dynamics of the male (**c**) and female (**d**) drosophila. No significant differences were observed between the standard diet (CK) and the treatment in any sample of the two groups.

We monitored the impact of the fecal microbiota exposure on the lifespan of the drosophila. The results showed that, compared to the control group, the average lifespan (30.73 + 2.78%) and the median lifespan (32 + 6.67%) of the treated male group were dramatically improved (*p* < 0.05); the average lifespan (32.81 + 9.73%) of the male F1 group was also significantly improved (*p* < 0.01) (Figure 6a). However, for female drosophila, the average lifespan (28.58 − 13.32%) and the median lifespan (29 − 12.12%) of the treated female group declined sharply (*p* < 0.05), while the lifespan of the female F1 group had only an inconspicuous change (average lifespan, 34.64 + 5.07% and median lifespan, 32 − 3.03%, *p* = 0.23) (Figure 6b). This finding was supported by the survival curve log-rank (i.e., Mantel–Cox) test (Table 1). In addition, the box plot showed that the fecal microbiota exposure did not have a significant impact on the body weight of the male and female drosophila (Figure 6c,d).

### 3.5. Effects of Fecal Microbiota Exposure on Vertical Climbing Ability and Expression of Age and Insulin-like Signaling-Related Genes of Drosophila

**Figure 7:**
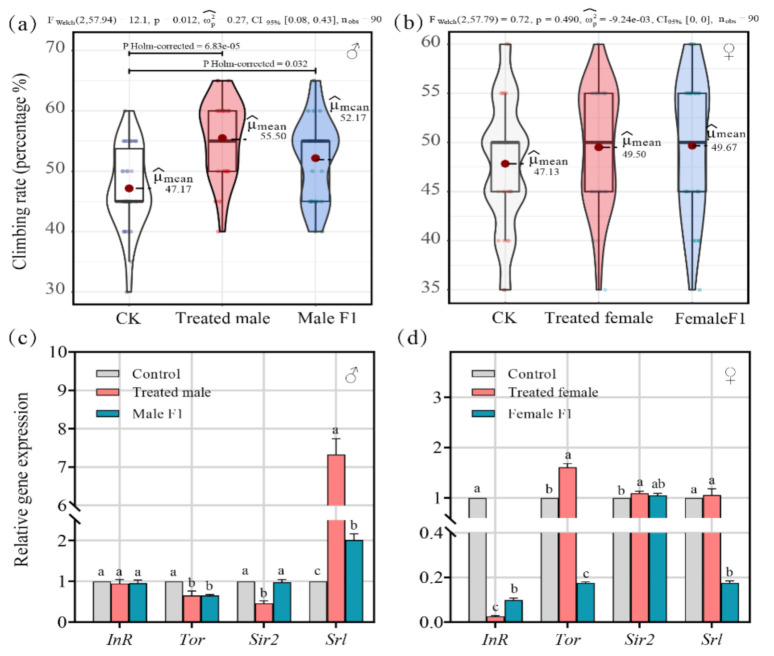
(**a**,**b**) Climb index shows how the treatment impacted the health conditions of the male (**a**) and female (**b**) drosophila; (**c**,**d**) 2. the effect of the treatment on the male (**c**) and female (**d**) drosophila based on relative gene transcript levels. Samples were taken from drosophila cultivated for 30 days.

All drosophila were subjected to strenuous activities to assess the impact of fecal microbiota exposure on their performance. After the fecal microbiota exposure, the vertical climbing ability of the treated male group (average μmean = 55.50) and the male F1 group (average μmean = 52.17) were significantly improved (average μmean = 47.17 in the control group) (*p* < 0.05) (Figure 7a), while the female drosophila showed no improvement (Figure 7b).

In addition, exploring the expression of genes that are closely related to the lifespan, growth, and development of drosophila explained how the physiology of drosophila could be affected by fecal microbiota exposure. The results show that fecal microbiota exposure affected gene expression patterns differently when comparing male and female drosophila (Figure 7c,d).

In the treated male group, the expression of the insulin-receptor-encoding gene (*InR*), which is related to growth and development in drosophila, was not significantly affected (*p* = 0.40) while the rapamycin-receptor gene (*Tor*) was dramatically downregulated (average of 0.66 times, *p* < 0.01). Sirtuin2 (*Sir2*) and spargel (*Srl*), which are linked with longevity in drosophila, were downregulated (average of 0.46 times, *p* < 0.01) and upregulated (average of 7.33 times, *p* < 0.01), respectively (Figure 7c).

In the male F1 group, the expression of *InR* (*p* = 0.37) and *Sir2* (*p* = 0.60) were not significantly affected (Figure 7c), but *Tor* and *Srl* were significantly decreased (average of 0.65 times, *p* < 0.01) and increased (average of 2.01 times, *p* < 0.01), respectively (Figure 7c).

In the treated female group, the expression of *InR* was downregulated (average of 0.03 times, *p* < 0.01); *Tor* and *Sir2* were both sharply upregulated (average of 1.61 times, *p* < 0.01; average of 1.10 times, *p* < 0.05), but *Srl* showed steady expression levels (*p* = 0.46) (Figure 7d).

In the female F1 group, the expression of *InR* (average of 0.99 times, *p* < 0.01), *Tor* (average of 0.18 times, *p* < 0.01), and *Srl* (average of 0.18 times, *p* < 0.01) were all downregulated, but *Sir2* expression did not show significant differences (*p* = 0.09) (Figure 7d).

## 4. Discussion

### 4.1. Effects of Fecal Microbiota Exposure on Drosophila Gut Bacteria

Similar to other humanized animal-model studies, the issue of imperfect transfer also appeared in this research. As compared to species of the Firmicutes phylum, the Actinobacteria and Bacteroides species from human donors showed higher abundances in male drosophila following exposure, indicating more successful transfer (Tables S5–S7). Meanwhile, the treated male group and the male F1 group showed F/B ratios that were close to that of the donor feces (Figure 2a). This is consistent with previous studies that transferred human fecal microbiota to mice [1,27,28]. All of these studies showed that Bacteroides rather than Firmicutes were selectively enriched by sterile mice [1,27,28]. Nonetheless, our treatment led to significant changes in the intestinal microbes of the drosophila. In our study, after thirty days of fecal exposure, the α-diversity of the intestinal bacteria in all of the tested drosophila was dramatically improved (Figure 1), which indicates that it more closely resembled the donor feces (average 2.54 in the Shannon index) (Tables S8 and S9). The sequencing analysis showed that 80–87.5% of the OTUs from the donor feces were adopted by the recipient drosophila during the 30 days of observation (Figure S1). In existing studies, the degree of mice humanization has been up to 75% of the human donor sequence mass [1]. We concluded that in this research an acceptable model with human microbiome was constructed. The results of subsequent analyses show that the male drosophila inherited more of the characteristics of the donor fecal microbiota. 

According to the results of the NMDS analysis (Figure 4d,e), we found that the fecal exposure impact on the drosophila intestinal microbiota was sex-dependent. In the early post-exposure period, the gut microbiota of both male and female groups was driven by the donor fecal microbiota, and the NMDS analysis showed significant beta distance in all of the groups at days 10 and 20 of observation (Figure 4d,e). The intestinal bacteria of the male and female drosophila subsequently became differentiated as measured on the 30th day (Figure 4d,e), where the intestinal bacteria of the male drosophila retained the characteristics of the donor fecal bacteria. However, the intestinal bacteria of the female drosophila more closely resembled the control samples. These findings indicate that the male drosophila could better adapt the bacteria from the donor feces.

Based on our research, this study may be the first to indicate that recruitment of intestinal bacteria is sex-dependent in drosophila. Han et al. reported that the gut microbial communities of drosophila were most strongly affected by the host strain, followed by the hostage and the host sex [29]. However, the drosophila showed a sex-dependent response to prebiotics, which had also been shown by Dong et al., namely that both 5% and 10% inulin increased the lifespan of male drosophila, as compared to the female, and the driving effect of inulin on the gut bacteria was also greater in male drosophila [21]. These results also provide evidence supporting the sex-dependent nature of bacterial recruitment in drosophila observed in this study.

The host-microbe interactions in drosophila are weak and continuous external infusions are needed to sustain their microbiome as endogenous maintenance is unreliable [17]. In addition, their intestines possess a defensive antimicrobial function that is activated when drosophila are exposed to environmental microorganisms, which include the production of antimicrobial peptides, reactive oxygen species, and a low pH environment by enterocytes [30]. All the microbes ingested by the drosophila through diet should also be screened. In this study, the male and female drosophila were cultured using a portion of the donor feces, but in separate confined spaces. Therefore, we speculate that the male and female drosophila may have different microbial defense and recruitment mechanisms, which may have resulted in the difference in the microbial componentization during the later stages of treatment (Tables S10 and S11). Collectively, our findings support the role of sex differences in forming gut microbiota in drosophila. Sex hormones appear to be responsible in part, but the pathways involved are unknown. This is consistent with the conclusions obtained in the studies of mammals. Early literature indicated that gut microbiota shows differentiation in mice of different sexes after puberty and male castration removed this trend [31]. Differences in gut microbiota composition between genders were clearly mediated at least in part by sex hormones [32]. Furthermore, the offspring of the drosophila inherited the response pattern of the donor feces from their parents. A significant difference was obtained from the beta distance between the female F1 group and the donor feces (Figure 2) in comparison to the male F1 group. This indicates that the male offspring of the drosophila could inherit more intestinal characteristics from the parental generation.

In addition, a previous study also indicated that the stable population in the gut of drosophila allowed a continuous bacterial delivery into the medium and was advantageous to the development of the next drosophila generation, particularly those rich in beneficial bacterium [33]. Regrettably, in our study, we did not monitor the microbes on the surface of the culture medium, nor did we investigate their role in the propagation process. Whether there were sex-dependent differences in this bacterial spreading mechanism should also be determined in future research.

Interestingly, we found that *Bifidobacterium*, a human-associated probiotic (average 70.13% in donor feces) had colonized in the male drosophila (average 21.19%) in this study. A higher ratio of *Bifidobacterium* in the donor fecal samples may be a result of the donor having taken probiotics for an extended period. The same trends were observed for another important probiotic, *Lactobacillus* (average 39.65% in the treated male drosophila). Although the female drosophila showed a high acceptance of these two probiotics (average 17.57% *Bifidobacterium* and 76.22% *Lactobacillus*), the bacterial communities of the female drosophila on the 30th sampling day is similar to that of the control group. This discovery that human-administered probiotic strains had colonized the drosophila gut may provide a basis for the application of drosophila with human microbiome in the study of differential performance and drug interactions resulting from probiotic strains present in the host.

### 4.2. Effects of Fecal Microbiota Exposure on Drosophila Lifespan and Body Weight

It has been reported that drosophila-associated microbes have profound effects on their physiology. Compared to the mammalian gastrointestinal tract, the drosophila melanogaster gut has some differences, but the overall structure and function are similar. Mammalian gut physiology and signaling pathways of gut development are highly conserved in drosophila melanogaster [34]. Mechanisms that limit contact between gut microbes and intestinal epithelial cells are also conserved in drosophila and mammals, including acid regions, mucus secretion, and secretion of antimicrobial peptides [16]. At the same time, there is growing evidence that gut-associated microbes have a strong influence on host traits, with drosophila longevity, behavior, the movement influenced by the composition of their gut microbiota [35]. Thus, in this study, the lifespan, body weight, vertical climbing ability, and growth and development of the drosophila were also taken as indicators to evaluate the impact of the donor fecal samples. 

The lifespans of the treated male and male F1 groups were significantly extended after fecal exposure while the lifespans of the treated female groups were significantly shortened (Figure 6a,b); however, the female F1 group was not significantly affected. Drosophila gut microbiota varied significantly with age, with strong expansion of most groups and a disproportionate increase in the abundance of pathogenic Gammaproteobacteria and *Enterococcus* species [36]. In our study, the over-enrichment of Gammaproteobacteria and *Enterococcus* was only observed in the control group (Figure 5a,b); in other words, they were non-selectively reduced in abundance after fecal exposure. This indicates that the change in intestinal bacteria may be the main reason for the extended lifespan of the male drosophila. The increase in the vertical climbing ability of the male drosophila also supports our speculation (Figure 7a,b).

It has been reported that different diets have inconsistent effects on the lifespans of male and female drosophila. Cinnamon was found to prolong the lifespan of male, but not female, drosophila [37]. Boyd et al. also reported sex-dependent effects of diet composition on drosophila lifespan; for example, in the treatment group with 4% peach extract, the average lifespan of the female drosophila was significantly increased, but no significant lifespan extension was found for male drosophila, while with 2%, the lifespans of the male and female drosophila were not significantly affected [38]. These studies indicate that nutritional and physiological needs related to sex may be responsible for the observed differences.

There is significant sexual dimorphism in the longevity mechanisms of drosophila. For example, in drosophila larvae, fasting induces a significant increase in *InR* gene expression in adult males, but not in females, and this affects their lifespan [39]. Westfall et al. proved that probiotic preparations could extend the lifespan of male drosophila by downregulating the expression of *InR* [40]. In our work, the downregulation of *InR* expression was observed, but it did not extend the lifespan of the female drosophila (Figure 6c,d). In the treated male and male F1 groups, the expression of *InR* was not significantly changed in comparison to the control group.

TOR kinase is a major amino acid and nutrient sensor that stimulates growth when food is sufficient. The inhibition of the TOR pathway has been shown to increase the lifespans of many species, including yeast [41], nematodes [42,43], mice [44], and drosophila [45]. The inhibition of dTOR activity can extend the average lifespan of drosophila by approximately 30% [45]. In this study, *TOR* expression in drosophila was inhibited in treated male and male F1 groups, which could have regulated the expression of downstream genes in the TOR signaling pathway to a certain extent and thereby prolonged the lifespan of the drosophila. However, *TOR* upregulation, which was observed in the treated female group, may have been responsible for their shortened lifespan.

Similar to its vertebrate counterpart PGC-1a, spargel (*Srl*) upregulates mitochondrial biogenesis and activity [46]. There was evidence that the overexpression of *Sir2* could extend the lifespan of drosophila [47], and *Srl* expression was related to the extension of their lifespans and physical abilities [48]. In this study, the upregulated expression of *Srl* was observed in treated male and male F1 samples, which was consistent with the significant increase in their vertical climbing rates (Figure 7a,c). It was also indicated that the extension of the male drosophila lifespan under fecal transferal may have a close relationship with the transcription level of *Srl*. In the treated female group, the expression of *Srl* was not consistent with the life curve (Figure 6b and Figure 7d). Therefore, we speculate that the colonization of the fecal microbiota may have caused sex-differentiated regulation of drosophila *Srl* expression, which could have partly led to the difference in lifespan dynamics. 

### 4.3. The Uses and Prospects of Anthropomorphic Drosophila Gut Model

In summary, the purpose of this study is to develop an anthropomorphic drosophila model of the gut microbiota, which could be applied in the gut microbe and host health studies. The gut microbes have become factors that must be considered for drug metabolism and bioaccumulation [49], and high-throughput in vivo models are helpful to study the interaction between drugs and intestinal bacteria. Compared with humanized murine models, our developed model has the advantages of rapid, high-volume reproduction with acceptable modeling accuracy. Despite the symbiotic system being constructed using wild-type drosophila and feces from healthy donors, the adaptability between host and donor feces suggested its potential in the development of more complex models. When donor microbiome from different humans with varying conditions are used, (e.g., diabetic, hypertense, and bipolar depression) then with different donors the potential for different roles of different bacteria might be observed. Mutant drosophila hosts (the human disease model) and donor feces with varying conditions (from patients) will be examined in the follow-up experiments.

Furthermore, due to the inconsistent performance of probiotics at the strain level, the biological functions of probiotics need to be screened strain by strain. In our study, two typical human-derived probiotics, *Bifidobacterium* and *Lactobacillus*, were shown to establish a good symbiotic relationship with the drosophila in this study, which indicates that the disease-related drosophila model could be a particularly suitable and attractive model for screening functional probiotics. The drosophila model proposed in this study provides new possibilities for the efficient execution of future research into human diseases, gut microbe, and drug interactions.

## 5. Conclusions

In this study, we have constructed a non-sterile drosophila model with human microbiome through antibiotic treatment and human fecal bacteria exposure. The treated drosophila maintained the humanized characteristics during the experimental period, and these were also partially retained in offspring male flies. The male drosophila showed a positive physiological response to the donor feces. Improved lifespan, climbing rate, mitochondrial biogenesis, and activity-related gene expression were obtained. Collectively, our study demonstrates a possibility to construct an anthropomorphic gut micro-ecosystem in drosophila. Methodological exploration in our study will provide a reference for establishing more disease-associated drosophila gut models in the future.

## Figures and Tables

**Table 1:** Statistics for survival curves. The total number of flies, mean and median lifespans (percentage changes), and log-rank (Mantel–Cox) test results in this study.

	Total n. of Flies	Mean (%Change)	Median (%Change)	Logrank (vs. Control)
Control	158	29.90	30	-
treated male	159	30.73 (+2.78%)	32 (+6.67%)	*p* < 0.05
male F1	160	32.81 (+9.73%)	30 (+0.00%)	*p* < 0.01
Control	158	32.97	33	-
treated female	159	28.58 (−13.32%)	29 (−12.12%)	*p* < 0.05
female F1	160	34.64 (+5.07%)	32 (−3.03%)	*p* = 0.23

## Supplementary Materials

The following supporting information can be downloaded at: https://www.mdpi.com/article/10.3390/microorganisms10030553/s1, Table S1: Primer Sequences of qRT-PCR used in this study, Table S2: qRT-PCR procedure setting, Figure S1: Venn diagram of OUT level, Table S3: Uninherited fecal genera in drosophila, Table S4: The exposure of fecal microbiota has enriched Male F1 with species that do not exist in the original intestinal bacteria of *Drosophila*, Table S5: Adonis analysis on genus level in treated male Drosophila, Table S6: Adonis analysis on genus level in treated female *Drosophila*, Table S7: Relative abundance of intestinal bacterial phyla in male *Drosophila* under the exposure of fecal microbiota, Table S8: Shannon index of male drosophila group, Table S9: Shannon index of female drosophila group, Table S10: Relative abundance of bacteria in fecal samples TOP 20 (male *Drosophila* group), Table S11: Relative abundance of bacteria in fecal samples TOP 20 (female *Drosophila* group).
