# Supplementary material for: Transfer of Human Microbiome to Drosophila Gut Model
Source: Microorganisms. 2022 Mar 3;10(3):553. doi: 10.3390/microorganisms10030553 (PMC8948740; doi:10.3390/microorganisms10030553)
Supplement: Supplementary file 1 [file microorganisms-10-00553-s001.zip › microorganisms-1570770-supplementary.pdf]

Table S1. Primer Sequences of qRT-PCR used in this study

Gene	Primer sequences (5'→3')	Reference
Sir2	CCGTTACTGAGGAGGAGCTG;<br>GTAGATCGCACACGTCCTTG	[1]
Srl	CTCTTGGAGTCCGAGATCCGCAA;<br>GGGACCGCGAGCTGATGGTT	[1]
InR	AACAGTGGCGGATTCGGTT;<br>TACTCGGAGCATTGGAGGCAT	[2]
Tor	GCTCAGAGGCGAGAGACAAG;<br>CCAGCTCACGGAGGATAAAG	[3]
Rp49	GACGCTTCAAGGGACAGTATCTG;<br>AAACGCGGTTCTGCATGA	[4]

Table S2. qRT-PCR procedure setting

Step	Time	Temperature
Hot start	2 min	95 °C
PCR reaction	15 s	95 °C
	25 s	55 °C
	35 s	72 °C
melting curve		1 °C / s

Figure S1. Venn diagram of OUT level

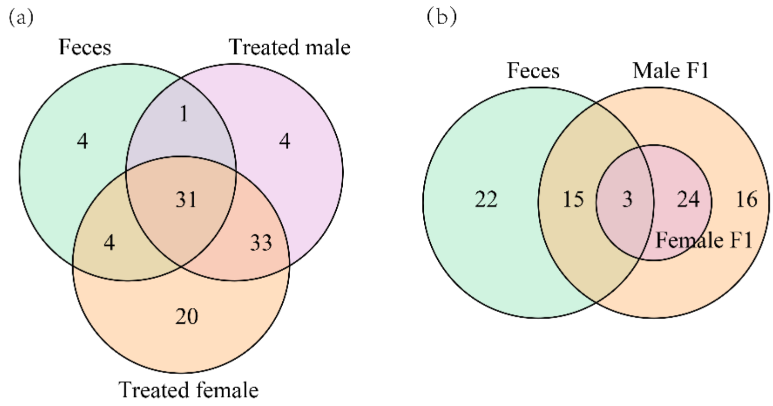

Table S3. Fecal genera that uncolonized in drosophila

**Uncolonized fecal genera in treated male group**		
	**Feces**	Male
Veillonella	0.1550%	0.0000%
Bacteroides	0.1133%	0.0000%
Ruminococcaceae UCG-013	0.0817%	0.0000%
uncultured bacterium	0.0133%	0.0000%
Neisseria	0.0017%	0.0000%
Uncolonized fecal genera in treated female group		
Species	Feces	Female
Clostridium sensu stricto 1	0.0217%	0.0000%
Ruminococcaceae UCG-013	0.0817%	0.0000%

Neisseria	0.0017%	0.0000%
Uncolonized fecal genera in female F1		
	Feces	Female F1
Bifidobacterium	76.9050%	0.0000%
Other	14.0300%	0.0000%
Lachnoclostridium	3.2267%	0.0000%
Subdoligranulum	0.6883%	0.0000%
Akkermansia	0.6650%	0.0000%
Escherichia-Shigella	0.4083%	0.0000%
uncultured bacterium	0.3083%	0.0000%
Veillonella	0.1550%	0.0000%
Bacteroides	0.1133%	0.0000%
Peptoclostridium	0.1100%	0.0000%
Intestinibacter	0.1017%	0.0000%
Ruminococcaceae UCG-013	0.0817%	0.0000%
Eggerthella	0.0467%	0.0000%
Erysipelatoclostridium	0.0267%	0.0000%
Clostridium sensu stricto 1	0.0217%	0.0000%
uncultured bacterium	0.0133%	0.0000%
Klebsiella	0.0117%	0.0000%
Proteus	0.0067%	0.0000%
Enterobacter	0.0050%	0.0000%
Neisseria	0.0017%	0.0000%
Uncolonized fecal genera in male F1		
	Feces	Female F1
Escherichia-Shigella	0.4083%	0.0000%
Veillonella	0.1550%	0.0000%
Peptoclostridium	0.1100%	0.0000%
Intestinibacter	0.1017%	0.0000%
Eggerthella	0.0467%	0.0000%
Erysipelatoclostridium	0.0267%	0.0000%
uncultured bacterium	0.0133%	0.0000%
Proteus	0.0067%	0.0000%
Neisseria	0.0017%	0.0000%

Table S4. The exposure of fecal microbes has enriched Male F1 with species that do not exist in the original intestinal bacteria of drosophila

**Species**	**Feces**	**CK**	**Male F1**
Subdoligranulum	0.6883%	0.0000%	0.0033%
Akkermansia	0.6650%	0.0000%	0.0083%
Bacteroides	0.1133%	0.0000%	0.0117%
Ruminococcaceae UCG-013	0.0817%	0.0000%	0.0067%
Clostridium sensu stricto 1	0.0217%	0.0000%	0.0017%
Klebsiella	0.0117%	0.0000%	0.0083%
Enterobacter	0.0050%	0.0000%	0.0033%

Table S5. Adonis analysis on genus level in treated male drosophila

	Df	Sums Of Sqs	Mean Sqs	F.Model	R2	Pr(>F)	Sig
Group	1	2.9790	1.48949	6.4819	0.32439	0.001	***
Residuals	27	6.2044	0.22979	0.67561			
Total	29	9.1834	1.00000				

Table S6. Adonis analysis on genus level in treated female drosophila

	Df	Sums Of Sqs	Mean Sqs	F.Model	R2	Pr(>F)	Sig
Group	1	0.5969	0.59694	2.9681	0.10612	0.036	*
Residuals	25	5.0280	0.20112	0.89388			
Total	26	5.6250	1.00000				

Table S7. Relative abundance of intestinal bacterial phyla in male drosophila under the exposure of fecal microorganisms

OTU ID	Feces	D10 CK	D20 CK	D30 CK	D10 Male Treated	D20 Male Treated	D30 Male Treated	Male F1
Actinobacteria	76.95%	0.01%	0.01%	0.00%	0.05%	0.12%	53.21%	0.02%
Bacteroidetes	0.42%	0.00%	0.00%	0.01%	0.00%	0.01%	0.60%	0.04%
Firmicutes	21.51%	1.51%	0.32%	1.21%	15.10%	2.86%	31.27%	0.20%

Table S8. Shannon index of male drosophila group

	Feces	D10 CK	D20 CK	D30 CK	D10 Male Treated	D20 Male Treated	D30 Male Treated	Male F1
shannon	2.54	0.81	0.80	0.87	2.35	2.80	3.61	1.15

Table S9. Shannon index of female drosophila group

	Feces	D10 CK	D20 CK	D30 CK	D10 Female Treated	D20 Female Treated	D30 Female Treated	Female F1
shannon	2.54	1.29	1.26	1.09	1.36	2.41	1.76	0.72

Table S10. Relative abundance of bacteria in fecal samples TOP 20 (male drosophila group)

OTU ID	D0	Abx	Feces	D10 CK	D20 CK	D30 CK	D10 Male Treated	D20 Male Treated	D30 Male Treated	Male F1
Bifidobacterium	0.01%	0.01%	76.91%	0.01%	0.00%	0.00%	0.03%	0.11%	21.12%	0.01%
Other	0.01%	0.17%	14.03%	0.00%	0.01%	0.00%	0.01%	0.02%	0.68%	0.03%
Lachnoclostridium	0.03%	0.59%	3.23%	0.00%	0.00%	0.00%	0.01%	0.13%	0.91%	0.01%
Enterococcus	8.84%	0.10%	2.63%	0.33%	0.01%	0.53%	11.04%	0.03%	2.35%	0.00%
Subdoligranulum	0.00%	0.00%	0.69%	0.00%	0.00%	0.00%	0.00%	0.00%	0.01%	0.00%
Akkermansia	0.00%	0.01%	0.67%	0.00%	0.00%	0.00%	0.00%	0.01%	0.95%	0.01%
Escherichia-Shigella	0.01%	0.12%	0.41%	0.00%	0.00%	0.00%	0.01%	0.05%	0.06%	0.00%
uncultured bacterium	0.00%	0.01%	0.31%	0.00%	0.00%	0.00%	0.00%	0.00%	0.37%	0.02%
Streptococcus	0.00%	0.09%	0.31%	0.00%	0.00%	0.00%	0.00%	0.02%	0.09%	0.05%
Veillonella	0.00%	0.04%	0.16%	0.00%	0.00%	0.00%	0.00%	0.00%	0.02%	0.00%
Lactobacillus	15.54%	11.93%	0.13%	1.16%	0.30%	0.66%	4.04%	2.63%	39.50%	0.04%
Bacteroides	0.00%	0.20%	0.11%	0.00%	0.00%	0.00%	0.00%	0.00%	0.18%	0.01%
Peptoclostridium	0.00%	0.00%	0.11%	0.00%	0.00%	0.00%	0.00%	0.00%	0.26%	0.00%

Intestinibacter	0.00%	0.00%	0.10%	0.00%	0.00%	0.00%	0.00%	0.00%	0.31%	0.00%
Ruminococcaceae UCG-013	0.00%	0.00%	0.08%	0.00%	0.00%	0.00%	0.00%	0.00%	0.00%	0.01%
Eggerthella	0.00%	0.00%	0.05%	0.00%	0.00%	0.00%	0.00%	0.00%	0.05%	0.00%
Erysipelatoclostridium	0.00%	0.03%	0.03%	0.00%	0.00%	0.00%	0.00%	0.00%	0.02%	0.00%
Clostridium sensu stricto 1	0.00%	0.02%	0.02%	0.00%	0.00%	0.00%	0.00%	0.00%	0.00%	0.00%
uncultured bacterium	0.00%	0.00%	0.01%	0.00%	0.00%	0.00%	0.00%	0.00%	0.01%	0.00%
Klebsiella	0.00%	0.00%	0.01%	0.00%	0.00%	0.00%	0.00%	0.00%	0.09%	0.01%

Table S11. Relative abundance of bacteria in fecal samples TOP 20 (female drosophila group)

OTU ID	D0	Abx	Feces	D10 CK	D20 CK	D30 CK	D10 Female Treated	D20 Female Treated	D30 Female Treated	Female F1
Bifidobacterium	0.00%	0.01%	76.91%	0.05%	0.01%	0.00%	0.71%	5.91%	17.57%	0.00%
Other	0.02%	0.79%	14.03%	0.00%	0.00%	0.00%	0.02%	0.13%	0.12%	0.00%
Lachnoclostridium	0.06%	2.74%	3.23%	0.00%	0.00%	0.00%	0.03%	0.35%	0.44%	0.00%
Enterococcus	13.06%	0.08%	2.63%	0.14%	0.79%	0.95%	1.68%	0.21%	0.43%	0.00%
Subdoligranulum	0.00%	0.00%	0.69%	0.00%	0.00%	0.00%	0.00%	0.00%	0.01%	0.00%
Akkermansia	0.00%	0.00%	0.67%	0.00%	0.00%	0.00%	0.03%	0.00%	0.01%	0.00%
Escherichia-Shigella	0.01%	0.33%	0.41%	0.00%	0.00%	0.00%	0.00%	0.09%	3.47%	0.00%
uncultured bacterium	0.00%	0.03%	0.31%	0.00%	0.00%	0.00%	0.00%	0.03%	0.03%	0.00%
Streptococcus	0.01%	0.23%	0.31%	0.02%	0.01%	0.00%	0.00%	0.04%	0.05%	0.00%
Veillonella	0.00%	0.00%	0.16%	0.00%	0.00%	0.00%	0.00%	0.00%	0.00%	0.00%
Lactobacillus	3.28%	1.27%	0.13%	14.95%	0.76%	0.32%	7.10%	20.81%	76.20%	0.03%
Bacteroides	0.00%	0.16%	0.11%	0.01%	0.00%	0.00%	0.00%	0.00%	0.00%	0.00%
Peptoclostridium	0.00%	0.00%	0.11%	0.00%	0.00%	0.00%	0.01%	0.11%	0.30%	0.00%
Intestinibacter	0.00%	0.01%	0.10%	0.00%	0.00%	0.00%	0.01%	0.10%	0.39%	0.00%
Ruminococcaceae UCG-013	0.00%	0.03%	0.08%	0.00%	0.00%	0.00%	0.00%	0.00%	0.00%	0.00%
Eggerthella	0.00%	0.00%	0.05%	0.00%	0.00%	0.00%	0.00%	0.01%	0.04%	0.00%
Erysipelatoclostridium	0.00%	0.13%	0.03%	0.00%	0.00%	0.00%	0.00%	0.01%	0.03%	0.00%
Clostridium sensu stricto 1	0.00%	0.01%	0.02%	0.00%	0.00%	0.00%	0.00%	0.07%	0.01%	0.00%
uncultured bacterium	0.00%	0.00%	0.01%	0.00%	0.00%	0.00%	0.00%	0.00%	0.00%	0.00%
Klebsiella	0.01%	0.00%	0.01%	0.00%	0.00%	0.00%	0.00%	0.00%	0.00%	0.00%

Reference

1. Tinkerhess, M.J.; Healy, L.; Morgan, M.; Sujkowski, A.; Matthys, E.; Zheng, L.; Wessells, R.J., The *Drosophila* PGC-1α homolog spargel modulates the physiological effects of endurance exercise. *PLoS ONE* **2012,** *7 (2)*, e31633.
2. Obata, F.; Fons, C.O.; Gould, A.P., Early-life exposure to low-dose oxidants can increase longevity via microbiome remodeling in *Drosophila*. *Nature Communications* **2018,** *9 (1)*, 975.
3. Boyd, O.; Weng, P.; Sun, X.; Alberico, T.; Laslo, M.; Obenland, D.M.; Kern, B.; Zou, S., Nectarine promotes longevity in *Drosophila melanogaster*. *Free Radical Biology and Medicine* **2011,** *50 (11)*, 1669-1678.
4. Storelli, G.; Defaye, A.; Erkosar, B.; Hols, P.; Royet, J.; Leulier, F., Lactobacillus plantarum promotes Drosophila systemic growth by modulating hormonal signals through TOR-dependent nutrient sensing. *Cell Metabolism* **2011,** *14 (3)*, 403-414.
